# Crystal Structure and Hirshfeld Surface Analysis of Acetoacetanilide Based Reaction Products

**DOI:** 10.3390/molecules25092235

**Published:** 2020-05-09

**Authors:** Farid N. Naghiyev, Jonathan Cisterna, Ali N. Khalilov, Abel M. Maharramov, Rizvan K. Askerov, Khammed A. Asadov, Ibrahim G. Mamedov, Khaver S. Salmanli, Alejandro Cárdenas, Ivan Brito

**Affiliations:** 1 Faculty of Chemistry, Baku State University, Z. Khalilov str. 23, Az 1148 Baku, Azerbaijan; amaharramov@bsu.edu.az (A.M.M.); rizvankam@bk.ru (R.K.A.); esedoglu@mail.ru (K.A.A.); ib.nmrlab@list.ru (I.G.M.); khavar_s.li@mail.ru (K.S.S.); 2 Departamento de Química, Universidad de Antofagasta, Avda. Universidad de Antofagasta 02800, Campus Coloso Antofagasta-Chile, Antofagasta 1240000, Chile; jonathan.cisterna@uantof.cl; 3 Department of Physics and Chemistry, “Composite Materials” Scientific Research Center, Azerbaijan State Economic University (UNEC), H. Aliyev str. 135, Az 1063 Baku, Azerbaijan; 4 Departamento de Física, Universidad de Antofagasta, Avda. Universidad de Antofagasta 02800, Campus Coloso Antofagasta-Chile, Antofagasta 1240000, Chile; alejandro.cardenas@uantof.cl

**Keywords:** acetoacetanilide, multicomponent reaction, X-ray analysis, Hirshfeld surface analysis

## Abstract

We report an unprecedented multicomponent reaction of acetoacetanilide with malononitrile leading to a structurally novel bicyclic product (9) in a high yield. The structure has been confirmed by X-ray crystallography and comparative Hirshfeld surface analysis of 5-cyano-2-hydroxy-2-methyl-*N*-phenyl-4-(yridine-4-yl)-6-(thiophen-2-yl)-3,4-dihydro-2*H*-pyran-3-carboxamide **2**, 5-cyano-2-hydroxy-2-methyl-6-oxo-*N*-phenyl-4-(thiophen-2-yl)piperidine-3-carboxamide **4** and 2-(8-amino-7,8a-dicyano-1-imino-4a-methyl-3-oxo-2-phenyl-1,3,4,4a,5,8a-hexahydroisoquinolin-6(2*H*)-ylidene)-*N*-phenylacetamide **9**.

## 1. Introduction

Pyridone, piperidine, pyran, and isoquinoline moieties are functional components of various natural products [1,2,3], pharmaceuticals [4,5,6], and biologically active compounds [7,8,9]. Two-component [10] and multicomponent [11,12,13] tandem transformations leading to these heterocyclic systems are particularly attractive from the point of view of diversity-oriented synthesis because of their modularity and rapid buildup of molecular complexity. In this article, we disclose a high-yielding synthesis of a structurally novel bicyclic heterocycle from two simple starting materials.

## 2. Results and Discussions

### 2.1. Chemical Context

**Scheme 1:**
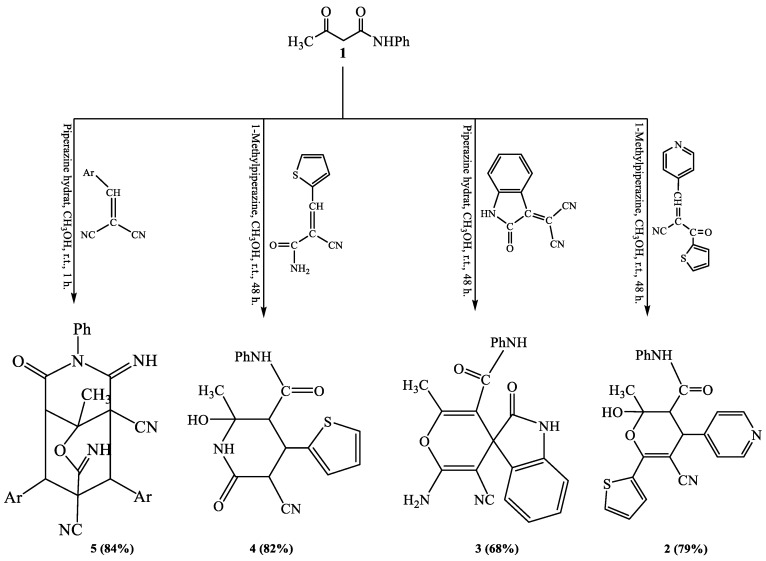
Previous works on acetoacetanilide **1** with various ylidenes [14,15,16,17].

In the context of our research program aimed at exploring the utility of acetoacetanilide **1** in multicomponent synthesis, we have recently reported a series of transformations illustrated in Scheme 1 [14,15,16,17]. Several double Michael acceptors bearing cyano groups at the α-position were found to give rise to structurally diverse heterocyclic products **2**–**5** when reacted with **1** in the presence of piperazine catalysts.

**Scheme 2:**
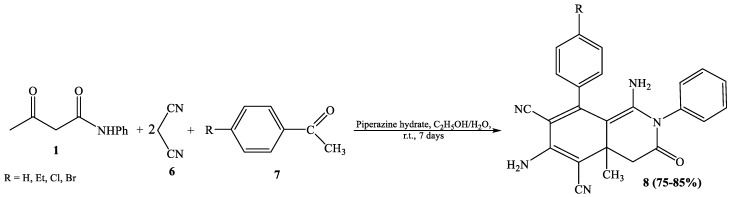
Previous work on acetoacetanilide **1** with malononitrile **6** and acetophenones **7** [19].

The benzylidenemalononitriles shown in Scheme 1 were prepared via the Knoevenagel reaction of aromatic aldehydes with malononitrile in catalyst-free media in EtOH/H_2_O in room temperature according to the reported procedure [18]. The analogous Knoevenagel reaction of acetophenones led to difficulties with isolation of the products. To circumvent this problem, we decided to combine the Knoevenagel step and subsequent reaction with acetoacetanilide and developed a one-pot synthesis of a series of bicyclic compounds **8** shown in Scheme 2 [19].

**Scheme 3:**
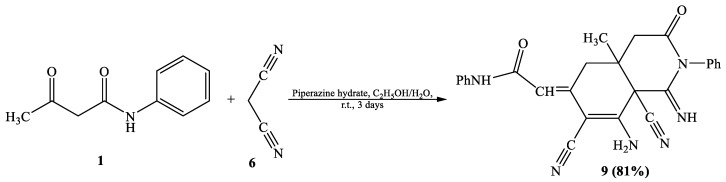
Reaction of acetoacetanilide **1** with malononitrile **6**.

Interestingly, the reaction of 4-aminoacetophenone (**7**, R=NH_2_) took a different course than other ketones examined. Instead of tetrahydroisoquinoline **8** we observed the formation of an unexpected hexahydroisoquinoline **9**, which did not incorporate 4-aminoacetophenone into its structure (Scheme 3). Evidently, the amino group deactivated the ketone carbonyl enough to preclude its participation in the condensation reaction. We confirmed that the new reaction proceeded equally well when 4-aminoacetophenone was omitted altogether from the reaction mixture (Scheme 3). 

**Scheme 4:**
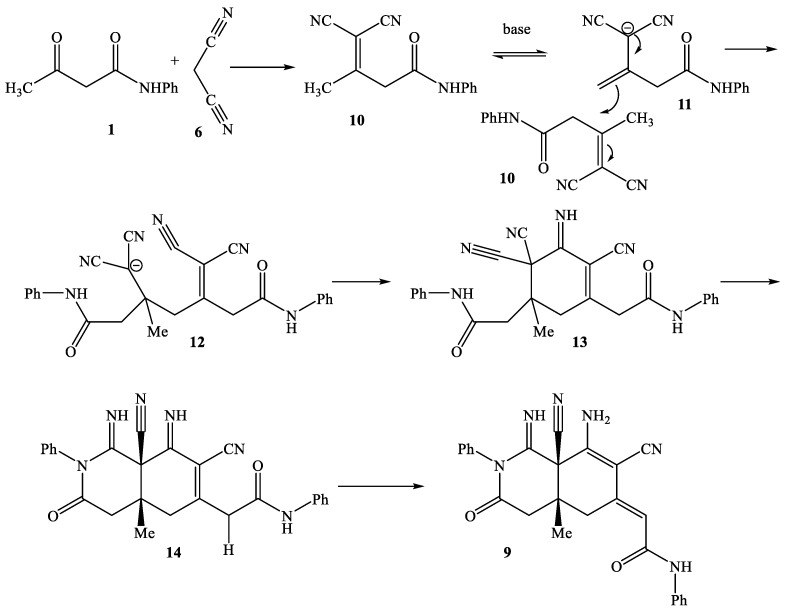
Proposed mechanism of formation of compound **9**.

The structure of title compound **9** indicates that it incorporates the elements of two molecules of acetoacetanilide and two molecules of malononitrile. To explain its formation, we propose the reaction pathway shown in Scheme 4. This tandem transformation starts with the Knovenagel condensation between the ketone carbonyl of **1** and malononitrile. The resulting product **10** undergoes base-catalyzed dimerization [20,21] to give stabilized carbanion **12**, which is set to add to the adjacent cyano group forming the carbocycle (cf. **13**). Another cyclization to give **14** is followed by tautomerization to the final product **9**. 

### 2.2. X-ray Analysis, Molecular, and Supramolecular Features and Hirshfeld Surface Analysis of ***2***, ***4***, and ***9***

**Figure 1:**
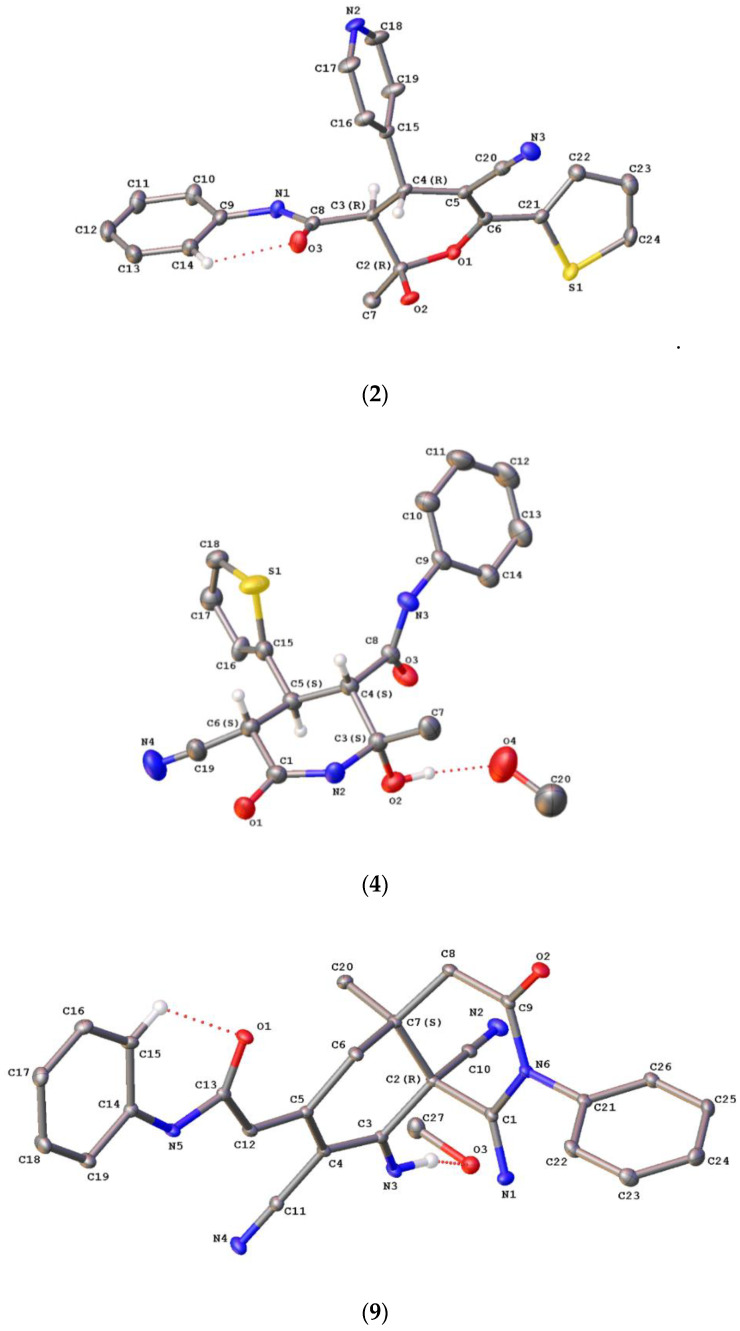
ORTEP plots for compounds **2**, **4**, and **9**. Thermal ellipsoids were drawn with 30% of probability. Some hydrogen atoms are removed for sake.

**Scheme 5:**
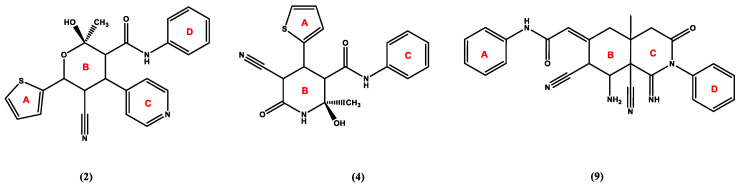
Ring plane defined in compounds **2**, **4**, and **9**.

The structure of compound **2**, consists of THP derivative with 2-thiophenyl, 4-pyridyl and *n*-phenyl acetamide, as an aromatic moiety attached to it. All substituents are attached in equatorial positions respect to the central THP ring. In the case of compound **4**, consists of 2-pyridone derivative with 2-thiophenyl, and *n*-phenylacetamide, as an aromatic moiety attached to it. All substituents are attached in equatorial positions respect to the central yridine ring. For compound **9**, the structure consists of hexahydroisoquinoline derivative with 1-phenyl and *n*-phenyl acetamide as an aromatic moiety attached to it. All substituents are attached in equatorial positions respect to the central hexahydroisoquinoline ring. All distances and bond angles are normal for all compounds [22]. All compounds have stereogenic centers and their relative absolute configurations are depicted in Figure 1. The molecules have a T-shaped (**2**) and V-shaped (**4** and **9**) form with the polysubstitued central rings as the junction point (Figure 1). According to Cremer & Pople parameters [23], the central rings for each compound have a semi-boat, for compound **2** (Q_T_ = 0.583(2) Å; θ = 124.3(2)°; ϕ = 267.2(3)°) and semi-chair conformation, for compounds 4 (Q_T_ = 0.506(2) Å; θ = 37.7(3)°; ϕ = 217.3(5)°) and 9 (Ring C2/C3/C4/C5/C6/C7: Q_T_ = 0.4991(18) Å; θ = 52.2(2)° ϕ = 305.7(3)°; Ring C1/C2/C7/C8/C9/N6: Q_T_ = 0.4638(18) Å; θ = 131.1(2)°; ϕ = 353.9(3)°). The analyzed torsion angles between all rings are depicted in Scheme 5 and Table 1.

**Figure 2:**
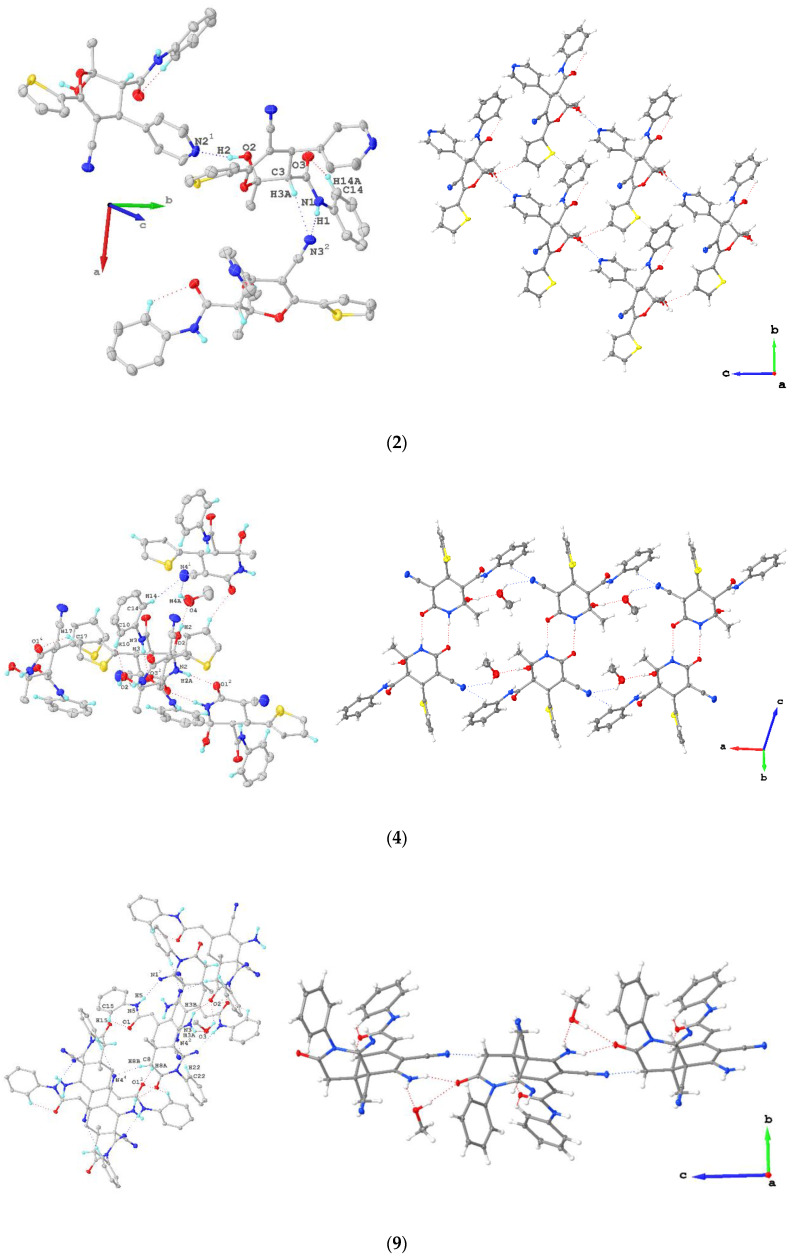
Intermolecular interactions observed in compounds **2**, **4** and **9**.

For compound **2**, one intramolecular C-H⋯O hydrogen bond interaction was found, where C–H moiety from phenyl ring acts as donor and O3 from the carbonyl group as acceptor from amide group. The crystal structure is stabilized by an extensive hydrogen-bonding linked by N–H⋯N(i); O–H⋯N(ii) and C–H⋯O(iii) with graph-set motif $C_{1}^{1}\left(9\right)$; $C_{1}^{1}\left(8\right)$; $C_{4}^{4}\left(32\right)$ and $R_{6}^{6}\left(48\right)$ forming 3D network [24], with base vector [100], [011] and [110] (Figure 2. Table 2). Another non-covalent weak interaction is also observed, specifically a chalcogen-π interaction between thiophenyl sulfur fragment and the phenyl ring, with ca. 3.6 Å, see Figure 2. 

For compound **4**,the crystal structure is also stabilized by an extensive hydrogen-bonding linked by N–H⋯O(iv and v); O–H⋯O(vi) and O–H⋯N(vii), where the methanol molecule participate with neighbor molecules forming different $D_{d}^{a}$ with graph-set motif $C_{1}^{1}\left(4\right)$; $C_{2}^{2}\left(10\right)$; $C_{4}^{4}\left(28\right)$; $R_{2}^{2}\left(8\right)$ and $R_{6}^{6}\left(36\right)$ forming 3D network [24], with base vector [010], [100] and [001] (Figure 2. Table 2). Another non-covalent weak interaction is also observed, specifically a hydrogen-π interaction between methyl group from piperidone ring and the phenyl ring, with ca. 3.2 Å, see Figure 2. 

For compound **9**, one bifurcated intermolecular C-H⋯O hydrogen bond interaction was found, where C6 and C15 acts as donors and O1 from the carbonyl group as acceptor. The crystal structure is also stabilized by an extensive hydrogen-bonding linked by O–H⋯O(viii); N–H⋯O(viii and ix), N–H⋯N(x), C–H⋯O(xi) and C–H⋯N(x and xii). The methanol molecule also participate with neighbor molecules forming different $D_{d}^{a}$ patterns, with graph-set motif $C_{4}^{4}\left(30\right)$ and $R_{6}^{6}\left(48\right)$, which include other $C_{d}^{a}$ and $R_{d}^{a}$ low level patterns. These aggregate forming 2D network [24], with base vector [010], [100] and [001], (Figure 2. Table 2). Another non-covalent interaction is also observed, specifically a dihydrogen bonding-type interaction between N1–H from hexaisoquinoline ring and C16/C17 of the phenyl ring, with ca. 3.0 Å, see Figure 2.

In order to visualize and verify intermolecular contacts across the crystal structure, the Hirshfeld surface analysis was made with complementary analyses such as shape index and curvedness surfaces.

**Figure 3:**
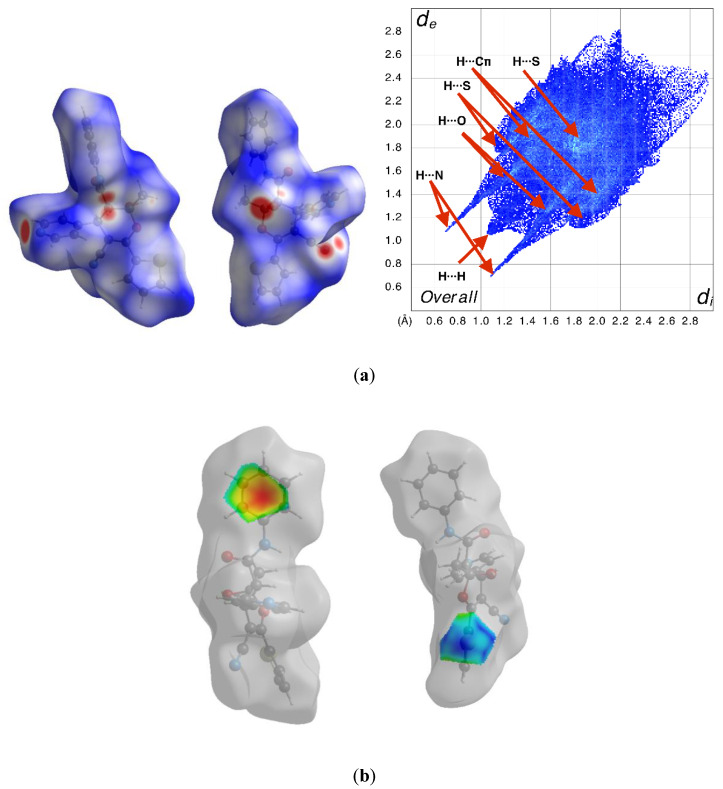
Hirshfeld surface analysis and fingerprint plot (**a**) and, Shape index maps (**b**) showing intermolecular interactions over compound **2**.

For compound **2**, the intermolecular interactions are mainly constituted by H⋯C, H⋯N and H⋯O, the contribution for both units are depicted in Figure 3. Where the reciprocal contacts appear as a broad wing for H⋯C, with 24.9% and *d*_e_ + *d*_i_ ≅ 3.1 Å, corresponding to H⋯π weak interactions. For H⋯N as a sharp needle with 18.1% and *d*_e_ + *d*_i_ ≅ 2.1 Å, when the distance is shorter than the VdW radii of N and H atoms (*d*_e_ + *d*_i_ < 2.75 Å) and, H⋯O as asymmetrical wings with 10.0% and *d*_e_ + *d*_i_ ≅ 2.6 Å. Another type of intermolecular contacts is also observed in the Hirshfeld Surface analyses. For example, in this compound, the contribution of H⋯S and C⋯S are around 5.9%, When *d*_e_ + *d*_i_ for each interaction are 2.9 and 3.6 Å, respectively and, their contributions are 5.1% and 3.8%, respectively. An C⋯S interaction is observed between thiophenyl and aromatic ring of the neighboor molecule being a chalcogen type interaction where the Sulphur atoms interact in the S⋯π pattern. To the best of our knowledge, this is one of first examples observed in the literature. The presence of this type of interaction is verified by shape index plot in the promolecule density (Figure 3).

For compounds **4** and **9**, the main contributions are classical hydrogen bond interaction when the MeOH solvent molecule helps to stabilize the crystal packing.

The contacts observed in compound 4 is a broad wing for H⋯C, with 22.9% and *d_e_* + *d_i_* ~ 2.6 Å, corresponding to H⋯π weak interactions in the crystal packing. For H⋯N as a sharp needle with 9.2% and *d_e_* + *d_i_* ~ 2.5 Å, and, H⋯O as symmetrical needle with 18.5% and *d_e_* + *d_i_* ~ 1.8 Å. Interestingly, in the analyses a very weak S⋯N interaction is also observed as symmetrical sharp needle with 1.8% and *d_e_* + *d_i_* ~ 3.5 Å, corresponding to heteroatomic chalcogen bond interaction in the crystal packing. 

**Figure 4:**
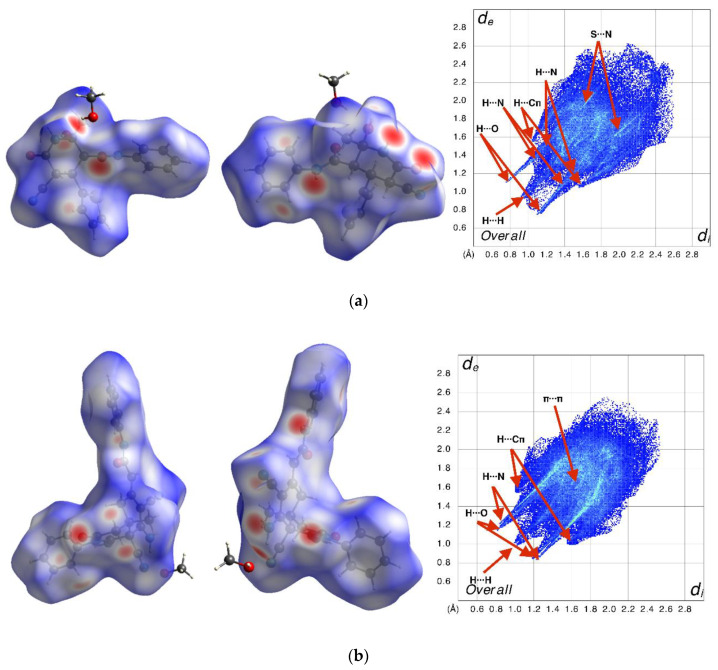
Hirshfeld surface analysis and fingerprint plot for compound **4** (**a**) and **9** (**b**).

For compound **9**, the contribution is a broad wing for H⋯C, with 16.5% and *d_e_* + *d_i_* ~ 2.6 Å, corresponding to H⋯π weak interactions in the crystal packing. For H⋯N as a sharp needle with 23.5% and *d_e_* + *d_i_* ~ 2.6 Å, and, H⋯O as symmetrical needle with 12.1% and *d_e_* + *d_i_* ~ 2.0 Å. moreover, in the analyses a very weak C⋯C interaction is also observed as symmetrical broad wings with 3.0% and *d_e_* + *d_i_* ~ 3.4 Å, corresponding to π⋯π interaction between aromatic ring parallel to *b*-axis (Figure 4).

In all cases, the interatomic contacts of H⋯H interactions generated 37.9, 42.6 and 43.0% of the Hirshfeld surface (Figure 3 and Figure 4) in compounds **2**, **4**, and **9** respectively, showing a broad triangular spot at diagonal axes *d_i_ + d_e_* ≅ 2.1 Å < 2.4 Å, denoting H⋯H short contacts with another significant effect on the molecular packing.

**Figure 5:**
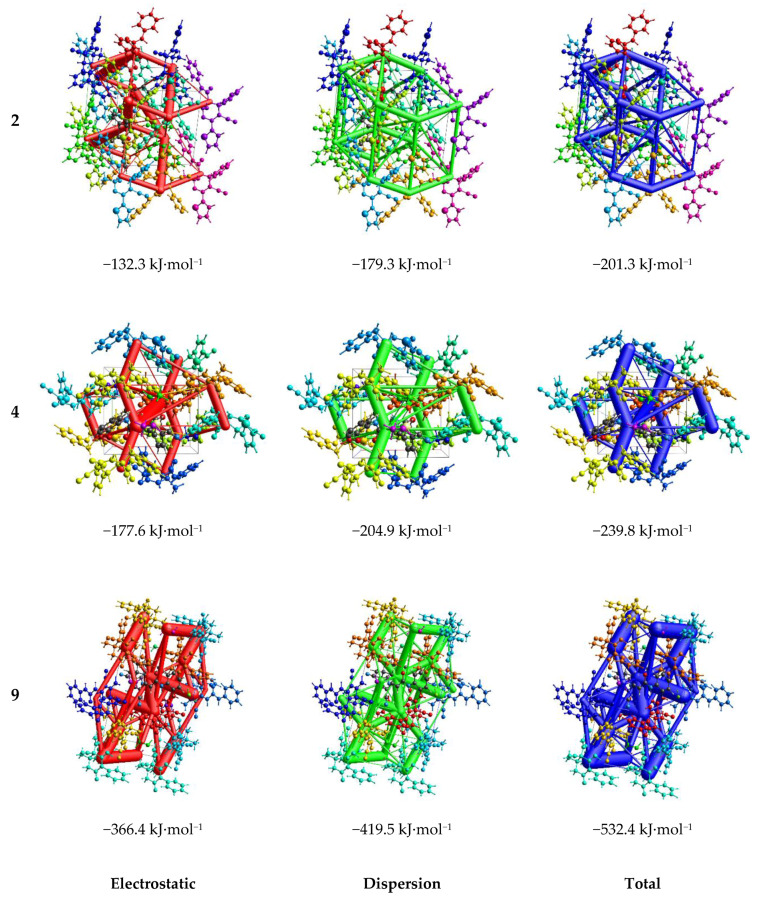
Energy framework diagrams for E_ele_, E_dis_ and E_tot_ for compounds **2**, **4**, and **9** showing the respective computed energies.

Finally, energy framework was analyzed to a better understanding of the packing and topology of crystal structure and the supramolecular rearrangement (Figure 5). According to the tube direction, it can conclude that the formation of the framework is directed by the translational symmetry elements in compound **2** and centrosymmetric setting for compounds **4** and **9**. This rearrangement allows the formation of another weak interactions in the crystal structure. The results of the calculations revealed that dispersion interactions exhibit approximately complex hexagonal shape energy topologies for compounds **2** and **9** and perpendicular ladders shape energy topologies for compound **4**. The small value of electrostatic energy versus dispersion energy in three compounds is attributed to the absence or few classical hydrogen bonds interactions. Despite several numbers of interactions in the crystal packing. 

## 3. Materials and Methods 

All chemicals were of reagent grade and used without further purification (Sigma-Aldrich, St. Louis, MO, USA). Melting points (mp’s) were recorded on a Stuart SMP30 melting point apparatus (Stuart-equipment, Staffordshire, UK) using open capillaries and were uncorrected. The NMR spectra were recorded at room temperature on a Bruker Avance II+ 300 spectrometer (Bruker Co.; Billerica, MA, USA) operating at 300 and 75 MHz for ^1^H and ^13^C, respectively. Chemical shifts were reported in ppm from tetramethylsilane using solvent resonance in DMSO-*d*_6_ solutions as the internal standard. 

### 3.1. Synthesis of ***2***, ***4***, and ***9***

Preparation of **2** and **4** was performed according to procedures reported in our previous papers [14,15]. Single crystals of title compounds were grown from ethanol solution by slow evaporation.

*2-(8-amino-7,8a-dicyano-1-imino-4a-methyl-3-oxo-2-phenyl-1,3,4,4a,5,8a-hexahydroisoquinolin-6(2H)-ylidene)-N-phenylacetamide* (**9**): To a 10.2 mmol solution of malononitrile in 20 mL of ethanol/water mixture (4/1 ratio) was added 10.2 mmol of acetoacetanilide and piperazine hydrate (7 mol%) and stirred in room temperature for 5 min. Immediate precipitation of colorless solid was observed, collected by filtration and was recrystallized in ethanol solution to get single crystals. Mp 177 °C, Yield 81%. ^1^H-NMR (75 MHz, DMSO-d_6_, δ): 1.44 (s, 3H, CH_3_); 2.80 (dd, 4H, 2CH_2_); 6.21 (s, 1H, CH=); 7.00–7.69 (m, 12H, 10CHarom+NH_2_); 8.10 (s, 1H, NH); 10.18 (s, 1H, NH=). ^13^C-NMR (75 MHz, DMSO-d_6_, δ): 24.19 (CH_3_), 34.77 (C_quart_), 34.89 (CH_2_), 39.25 (CH_2_), 53.32 (C_quart_), 80.29 (=C_quart_), 113.87 (CH=), 115.85 (CN), 116.54 (CN), 119.47 (2CH_arom_), 123.51 (CH_arom_), 129.16 (3CH_arom_), 129.59 (2CH_arom_), 130.07 (CH_arom_), 130.61 (CH_arom_), 134.08 (C_ar_.), 139.98 (C_ar_.), 142.58 (=C_quart_), 152.49 (=C_quart_), 153.49 (N−C=O), 165.05 (N−C=NH), 167.34 (N−C=O). 

### 3.2. X-ray Structure Determination

Some suitable crystals of compounds **2**, **4**, and **9** were measured and their diffraction data were collected at 296 K for compound **2** and **4**. Meanwhile, **9** was measured at 100K, on Bruker APEXII CCD diffractometer (Bruker Co.; Billerica, MA, USA). The diffraction frames were integrated using the APEX2 package [25] and were corrected for absorptions with SADABS. The structure of **2**, **4**, **9** were solved by intrinsic phasing using the OLEX2 program [26]. All the structures were then refined with full-matrix least-square methods based on *F^2^* (SHELXL-2015) [27]. For the three compounds, non-hydrogen atoms were refined with anisotropic displacement parameters. All hydrogen atoms were included in their calculated positions, assigned fixed isotropic thermal parameters and constrained to ride on their parent atoms. A summary of the details about crystal data, collection parameters, and refinement are documented in Table 3, and additional crystallographic details are in the CIF files. ORTEP views were drawn using OLEX2 software [26]. Aditionally, Atomic coordinates, bond lengths, bond angles and thermal parameters have been deposited at the Cambridge Crystallographic Data Centre (CCDC). These data can be obtained free of charge via www.ccdc.cam.uk/conts/retrieving.html (or from the CCDC, 12 Union Road, Cambridge CB2 1EZ, UK; fax: +44-1223-336033; or deposit@ccdc.cam.ac.uk). Any requests to the CCDC for data should quote the full literature citation and CCDC reference numbers for **2**, **4**, and **9** 1875230, 1878204, 1882582 respectively.

### 3.3. Computations

Intermolecular interactions have been computed with Crystalexplorer 17.5 [28] using Hirshfeld surface analysis [29] and two dimensional fingerprint plots [30]. The *d_norm_* plot was estimated via calculations of the external (*d_e_*) and internal (*d_i_*) distances to the nearest nucleus (*d_i_* + *d_e_*). Energy frameworks were computed using the 6–31G (d,p) basis set at B3LYP functional [31,32]. To deal with the positional disorder in compounds **2** and **4**, this was modeled using Rigid body restrain (RIGU), using major occupancy factor part in each compound (0.790 and 0.585 for compounds **2** and **4**, respectively) fixing final occupancy with 1.0, to compute the molecular surface in the crystal structure without further problems. 

## 4. Conclusions

Finally, we summarized our previous works about the reaction of acetoacetanilide with ylidenes and report the unexpected formation of hexahydroisoquinolin derivative. Detailed X-ray and Hirshfeld Surface analysis of three title compounds was done in the presented work.

## Figures and Tables

**Table 1:** Torsion angles (°) * of compounds **2**, **4**, and **9**.

Plane\Compound	(2)	(4)	(9)
AB	26.2(2)	98.7(3)	18.37(6)
AC	53.2(2)	167.1(7)	25.21(11)/76.98(6)[f]
AD	101.4(3)	-	63.01(7)/54.69(7)[f]
BC	75.58(11)	57.17(10)	49.67(7)/58.60(7)[f]
BD	104.1(11)	-	108.65(10)/102.43(7)[f]
CD	23.2(3)	-	73.76(6)

* Defined by twist angle in the plane of scheme above; [f]= fold angle between rings.

**Table 2:** Hydrogen bond interactions for compounds **2**, **4**, and **9**.

**Compound 2**						
**D**	**H**	**A**	**d(D-H)/Å**	**d(H-A)/Å**	**d(D-A)/Å**	**D-H-A/°**
O2	H2	N2 ^1^	0.879(4)	1.792(10)	2.653(3)	166(4)
N1	H1	N3 ^2^	0.79(3)	2.44(3)	3.223(4)	167(3)
C3	H3A	N3 ^2^	0.98	2.44	3.368(3)	157.9
C14	H14A	O3	0.93	2.34	2.925(4)	120.5
Symmetry codes: ^1^ 1/2−X, −1/2+Y, −1/2+Z; ^2^ 1/2+X, 1/2−Y, +Z						
**Compound 4**						
**D**	**H**	**A**	**d(D-H)/Å**	**d(H-A)/Å**	**d(D-A)/Å**	**D-H-A/°**
O4	H4A	N4 ^1^	0.880(3)	2.38(16)	3.059(5)	134(19)
O2	H2	O4	0.881(3)	1.960(11)	2.822(4)	166(4)
N2	H2A	O1 ^2^	0.89(3)	2.01(3)	2.893(3)	175(2)
N3	H3	O3 ^3^	0.84(3)	2.04(3)	2.864(3)	167(3)
C10	H10	O2 ^3^	0.93	2.79	3.389(4)	123.6
C14	H14	N4 ^1^	0.93	2.70	3.438(5)	136.5
C17	H17	O1 ^4^	0.93	2.48	3.190(7)	132.8
C18′	H18′	O1 ^4^	0.93	2.73	3.365(9)	126.3
Symmetry codes: ^1^ −1+X, +Y, +Z; ^2^ 1−X, 1-Y, 1−Z; ^3^ 1/2−X, 1/2+Y, 3/2−Z; ^4^ 3/2−X, −1/2+Y, 3/2−Z						
**Compound 9**						
**D**	**H**	**A**	**d(D-H)/Å**	**d(H-A)/Å**	**d(D-A)/Å**	**D-H-A/°**
O3	H3	O2 ^1^	0.92(3)	2.46(3)	3.0847(18)	125(2)
N3	H3B	O3	0.91(2)	2.06(2)	2.856(2)	146.6(17)
N3	H3A	O2 ^1^	0.88(2)	2.13(2)	2.8616(18)	139.6(17)
N5	H5	N1 ^2^	0.828(19)	2.24(2)	3.059(2)	172.6(18)
C8	H8A	O1 ^3^	0.99	2.30	3.202(2)	150.3
C8	H8B	N4 ^4^	0.99	2.48	3.448(2)	166.3
C15	H15A	O1	0.95	2.28	2.879(2)	120.0
C22	H22A	N4 ^2^	0.95	2.60	3.551(2)	174.1
Symmetry codes: ^1^ +X, 3/2−Y, −1/2+Z; ^2^ 1−X, 1−Y, 1−Z; ^3^ 1−X, 1/2+Y, 3/2−Z; ^4^ +X,3/2−Y, 1/2+Z						

**Table 3:** Crystal data parameters for compounds **2**, **4**, and **9**.

Compound	2	4	9
**Empirical Formula**	C_23_H_19_N_3_O_3_S	C_19_H_21_N_3_O_4_S	C_27_H_26_N_6_O_3_
**Formula Weight**	417.47	387.45	482.54
**Temperature/K**	296(2)	296(2)	100(2)
**Crystal System**	Orthorhombic	Monoclinic	Monoclinic
**Space Group**	Pna2_1_	P2_1_/n	P2_1_/c
**a/Å**	12.7792(3)	10.4527(5)	13.5513(6)
**b/Å**	11.3102(2)	9.4679(5)	9.8594(5)
**c/Å**	14.8019(3)	19.4440(9)	18.2460(11)
**α/°**	90	90	90
**β/°**	90	98.956(2)	102.003(2)
**γ/°**	90	90	90
**Volume/Å^3^**	2139.40(8)	1900.82(16)	2384.5(2)
**Z**	4	4	4
**ρ_calc_g/cm^3^**	1.296	1.354	1.344
**μ/mm^−1^**	0.180	0.200	0.091
**F(000)**	872.0	816.0	1016.0
**Crystal Size/mm^3^**	0.546 × 0.279 × 0.196	0.274 × 0.198 × 0.122	0.210 × 0.175 × 0.110
**Radiation Å**	MoKα (λ = 0.71073)	MoKα (λ = 0.71073)	MoKα (λ = 0.71073)
**2Θ Range for Data Collection/°**	4.532 to 57.978	4.178 to 55.988	4.564 to 51.994
**Index Ranges**	−17 ≤ h ≤ 17, −15 ≤ k ≤ 15, −20 ≤ l ≤ 20	−13 ≤ h ≤ 13, −12 ≤ k ≤ 12, −25 ≤ l ≤ 25	−15 ≤ h ≤ 16, −12 ≤ k ≤ 12, −22 ≤ l ≤ 22
**Reflections Collected**	29818	24793	23205
**Independent Reflections**	5626 [R_int_ = 0.0348, R_sigma_ = 0.0274]	4550 [R_int_ = 0.0668, R_sigma_ = 0.0536]	4520 [R_int_ = 0.0620, R_sigma_ = 0.0504]
**Data/Restraints/Parameters**	5626/215/317	4550/66/300	4520/0/347
**Goodness-of-Fit on F^2^**	1.005	1.031	1.002
**Final R Indexes [I ≥ 2σ (I)]**	R_1_ = 0.0432, wR_2_ = 0.0994	R_1_ = 0.0677, wR_2_ = 0.1599	R_1_ = 0.0486, wR_2_ = 0.0930
**Final R Indexes [all data]**	R_1_ = 0.0504, wR_2_ = 0.1027	R_1_ = 0.1214, wR_2_ = 0.1803	R_1_ = 0.0747, wR_2_ = 0.0996
**Largest Diff. Peak/Hole/e Å^−3^**	0.22/−0.18	0.24/−0.32	0.24/−0.21
**Flack Parameter**	0.02(2)	-	-
